# Occupational radiation exposure assessment during the management of [^68^Ga]Ga-DOTA-TOC

**DOI:** 10.1186/s40658-022-00505-8

**Published:** 2022-10-29

**Authors:** Mercedes Riveira-Martin, Lara Struelens, Werner Schoonjans, Isaac Sánchez-Díaz, Jose Muñoz Iglesias, Óscar Ferreira Dávila, Francisco Javier Salvador Gómez, Manuel Salgado Fernández, Antonio López Medina

**Affiliations:** 1 Medical Physics and RP Department, Galicia Sur Health Research Institute, Vigo, Spain; 2 Belgian Nuclear Research Centre (SCK CEN), Mol, Belgium; 3 Nuclear Medicine Department (SERGAS), University Hospital of Vigo, Meixoeiro Hospital, Vigo, Spain; 4 Medical Physics and RP Department (GALARIA), University Hospital of Vigo, Meixoeiro Hospital, Vigo, Spain

**Keywords:** Nuclear medicine, Occupational exposure, Extremity dosimetry, Equivalent dose, Effective dose, [^68^Ga]Ga-DOTA-TOC

## Abstract

**Background:**

Since it was first approved in Europe in 2016, the gallium-68 (^68^Ga) radiopharmaceutical [^68^Ga]Ga-DOTA-TOC has been widely used for imaging of somatostatin receptor (SSTR) positive tumours using positron emission tomography–computed tomography (PET/CT). Significant patient benefits have been reported, so its use is rapidly increasing. However, few studies have been published regarding occupational doses to nuclear medicine personnel handling this radiopharmaceutical, despite its manual usage at low distances from the skin and the beta-emission decay scheme, which may result in an increased absorbed dose to their hands. In this context, this study aims to analyse the occupational exposure during the administration of [^68^Ga]Ga-DOTA-TOC for PET/CT imaging. For this purpose, extremity, eye lens and whole-body dosimetry in terms of *Hp(0.07)*, *Hp(3)* and *Hp(10)*, respectively, was conducted on six workers with both thermoluminescent dosimeters, and personal electronic dosimeters.

**Results:**

The non-dominant hand is more exposed to radiation than the dominant hand, with the thumb and the index fingertip being the most exposed sites on this hand. Qualitative analysis showed that when no shielding is used during injection, doses increase significantly more in the dominant than in the non-dominant hand, so the use of shielding is strongly recommended. While wrist dosimeters may significantly underestimate doses to the hands, placing a ring dosimeter at the base of the ring or middle finger of the non-dominant hand may give a valuable estimation of maximum doses to the hands if at least a correction factor of 5 is applied. Personal equivalent doses for the eyes did not result in measurable values (i.e., above the lowest detection limit) for almost all workers. The extrapolated annual dose estimations showed that there is compliance with the annual dose limits during management of [^68^Ga]Ga-DOTA-TOC for diagnostics with PET in the hospital included in this study.

**Conclusions:**

Imaging with [^68^Ga]Ga-DOTA-TOC is a safe process for the workers performing the administration of the radiopharmaceutical, including intravenous injection to the patient and the pre- and post-activity control, as it is highly unlikely that annual dose limits will be exceeded if good working practices and shielding are used.

**Supplementary Information:**

The online version contains supplementary material available at 10.1186/s40658-022-00505-8.

## Background

The increasing number of Nuclear Medicine (NM) procedures, as well as recent and pending approvals of novel diagnostic and therapeutic radiopharmaceuticals, lead to an increase in the number of workers in NM departments, the types of examinations and therapies, and the number of patients undergoing these procedures [1, 2]. Despite promising patient outcomes, this trend inevitably increases the exposure of workers to radiation sources. In addition, NM professionals work in proximity to sealed and unsealed radioactive sources and radiation equipment, resulting in a high risk of irradiation, especially to the hands. Therefore, it is important to assess the dose received by workers due to these procedures, which require the manual use of high-activity sources at low distances from the skin [3–5] which may increase the risk of exposure. This practice requires monitoring of the radiation dose to the skin on the fingers, wrists, eye lens and chest, where the exposure is likely to be the highest [6].

To properly assess the doses received by workers and to prevent exceeding the recommended limits, an evaluation of the doses received is performed on a monthly or annual basis by means of personal passive detectors, such as ring or wrist dosimeters, which are common in daily clinical practice in Spain. However, the high gradient with which doses are deposited on the hands [7, 8], particularly in the case of the handling of beta sources, hinders the monitoring of exposure on the hands, since as the fingertips are commonly more exposed, ring and wrist dosimeters usually underestimate the dose. Therefore, according to the ORAMED project [6] an appropriate correction factor is needed when using these devices. Nevertheless, the ORAMED project, which focused on optimising radiation protection of medical staff, was limited to ^99m^Tc and ^18^F-labelled radiopharmaceuticals as well as ^90^Y in peptide receptor radiotherapy (PRRT) and radioimmunotherapy (RIT), and many new applications in NM have been introduced since then, such as positron emission tomography/computed tomography (PET/CT) examinations using gallium-68-labelled radiopharmaceuticals.

The use of gallium-68 (^68^Ga) has increased over the last few years [9], especially for the diagnosis of prostate cancer (PCa) with [^68^Ga]Ga-PSMA [10], and neuroendocrine tumours (NET) with [^68^Ga]Ga-DOTA-TOC [11, 12]. However, these are still novel radiopharmaceuticals, so there is a paucity of studies addressing occupational dosimetry from exposure to ^68^Ga-based peptides [13]. Furthermore, even though the activities administered during other procedures based on common radionuclides, such as ^18^F, may be within the same order of magnitude as with ^68^Ga, the maximum energy of the ^68^Ga decay particles (1.899 MeV) is considerably higher than of ^18^F (0.634 MeV), which means that the radiation exposure to ^68^Ga-labelled radiopharmaceuticals should be studied thoroughly and individually [13]. This is especially relevant when it comes to extremity dosimetry, since as a positron emitter, handling a ^68^Ga-based radiopharmaceutical at short distances can considerably increase the absorbed dose to the skin of the hands [14]. According to a recent survey among national dose registries, performed by Kyriakidou et al. [15], there are still large variations in the methods of dosimetry and determination of the maximum dose to extremities in NM departments. This leads to the need to arbitrate methods to reduce the uncertainty in the estimation of NM doses, and to facilitate comparisons between different dosimetry results.

According to a literature review performed by Kollaard et al. [13], at the time there was only one publication reporting on extremity doses for ^68^Ga [16], but presented a practice without source shielding, which was not representative of ongoing practices, and was focused on another peptide, [^68^Ga]Ga-DOTA-NOC. To the best of our knowledge, few more studies have been published since then [11, 17] but no specific extremity, eye lens dosimetry nor real-time monitoring was performed.

In addition, the use of the PET tracer [^68^Ga]Ga-DOTA-TOC deserves special attention because it is not only suitable for imaging, but also for theranostic applications in combination with the peptide [^177^Lu]Lu-DOTA-TATE, mainly for treating NETs with PRRT, despite having different binding peptides [18]. In this context, dosimetry studies involving exposure to both [^68^Ga]Ga-DOTA-TOC and [^177^Lu]Lu-DOTA-TATE radiopharmaceuticals must be performed, and preliminary results have already been presented [19]. Nevertheless, as a first approach in this work we focused on the former peptide for diagnostics, the preliminary study having been presented and accepted as an oral presentation at the 35th Annual Congress of the European Association of Nuclear Medicine (EANM) [20].

This research is part of the SINFONIA research project (supported by the European Commission within the Euratom research and training programme 2019–2020 [21]). This study aims to address the scarcity of studies concerning occupational doses during the management of the diagnostic radiopharmaceutical [^68^Ga]Ga-DOTA-TOC by assessing the radiation exposure to extremities, eye lenses and whole-body of professionals working in our NM Department during these procedures. For this purpose, different types of passive and active dosimeters were used. Doses were reported in terms of the personal equivalent dose *Hp(3)* to the eyes, the personal effective dose *Hp(10)* to the whole-body, and the personal equivalent dose *Hp(0.07)* to the extremities. Besides, a comparison between the dose recorded with the common ring and wrist dosimeters used in routine practice and the exposure to specific monitoring positions on the fingers was performed in order to set a proper correction factor, as suggested by the ORAMED project [6]. Finally, annual extrapolations were performed to verify compliance with the regulatory prescribed dose limits.

## Methods

### Radionuclide of study

This study is focused on the radioactive exposure during the administration of [^68^Ga]Ga-DOTA-TOC (^68^ Ga-DOTA-D-Phe1-Tyr3-Octreotide or ^68^Ga-DOTATOC), an amino acid peptide bounded to the chelator DOTA and labelled with the radionuclide ^68^Ga. The radionuclide ^68^Ga is a positron emitter (β^+^) that decays with a half-life of 67.7 min to the stable isotope zinc-68 (^68^Zn). The maximum *β*+ energy is 1899 keV (average 836 keV) and the positron yield is 89.1%. The mean positron range before annihilation with an electron is 1.05 mm in soft tissue [22].

The ^68^Ga labelling takes place in an external facility with a preparation kit for ^68^Ga-labeling of DOTA-TOC (SomaKit TOC®, AAA, a Novartis company, Saint-Genis-Pouilly, France) with ^68^Ge/^68^Ga generators. It is administered intravenously within 4 h of labelling, with activities ranging from 100 to 260 MBq per injection, in compliance with the 2017 EANM procedural guideline on PET/CT tumour imaging with ^68^Ga-DOTA-conjugated peptides [23]. The estimated effective dose from the administration of 200 MBq activity to a 70 kg adult is approximately 4.2 mSv. ^68^Ga-DOTATOC is indicated for PET imaging of somatostatin receptor overexpression in adult patients with confirmed or suspected well-differentiated gastroenteropancreatic neuroendocrine tumours (GEP-NET) to localize primary tumours and their metastases.

### Monitored staff and radiopharmaceutical administration

The study was conducted over a period of 11 months at Meixoeiro Hospital (Spain) and encompassed a total of 28 patients who were administered ^68^Ga-DOTATOC for PET/CT imaging, each patient being injected once. A total of six workers (nurses), all being right-handed, were monitored while manipulating the radiopharmaceutical, which is administered by a single nurse per patient. From nurses 1 to 6, the cumulated dose over 5, 6, 4, 6, 6, and 1 sessions was recorded, respectively (i.e., a total of 28 sessions, imaging one patient per session). The total activity handled over all monitored sessions was obtained from the syringe activity measured in each one. This information is summarized in Table 1, with values presented as mean ± standard deviation (SD).

**Table 1:** Summary of the data recorded over the sessions in which each worker was monitored

	Syringe shielding	Monitored sessions	Activity (GBq)	Syringe volume (ml)	Initial activity (MBq)	Residual activity (MBq)	Time (s)
Nurse 1	W	5	0.85	3.6 ± 0.4	170.5 ± 50.5	10.0 ± 8.3	120.0 ± 0.0
Nurse 2	W	6	1.31	3.5 ± 0.3	218.9 ± 43.5	6.6 ± 3.4	132.0 ± 37.5
Nurse 3	No^a^	4	0.84	3.8 ± 0.5	210.9 ± 68.8	9.4 ± 4.5	120.0 ± 0.0
Nurse 4	W	6	1.11	4.4 ± 0.4	184.8 ± 8.6	8.6 ± 1.5	140.2 ± 22.1
Nurse 5	W	6	1.20	4.5 ± 0.3	199.6 ± 24.8	8.0 ± 1.8	176.5 ± 44.9
Nurse 6	W	1	0.25	4.2 ± –	252.7 ± –	7.2 ± –	180.0 ± –
Mean ± SD				4.0 ± 0.5	199 ± 42	8.3 ± 3.9	141.1 ± 20.9

Values are referred to as mean ± SD

^a^Only during injection

The intravenous injection of the radiopharmaceutical is performed with the prefilled syringe containing a mean of 4.0 ± 0.5 ml (range 3.0–4.8 ml) yielding an activity per patient of 199 ± 42 MBq (range 135.4–310.8 MBq). The residual activity in the syringe is measured with the activimeter at the end of each session, with an average of 8.3 ± 3.9 MBq (range 0.9–24.8 MBq).

**Fig. 1:**
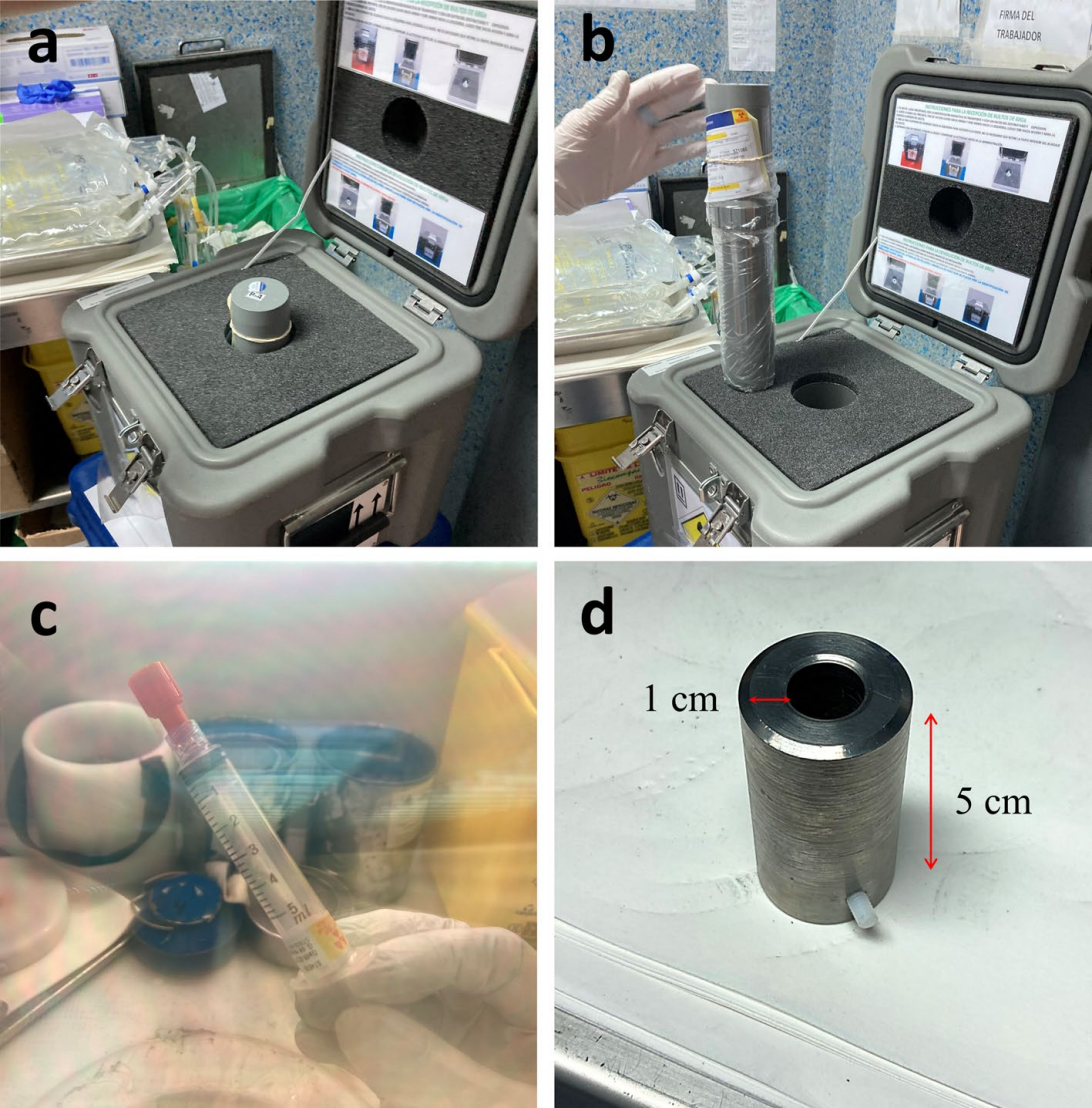
Material used for the administration of ^68^Ga-DOTATOC: **a** Shielded container, **b** PMMA cylinder shielding the syringe, **c** interior of the hot cell seen through the lead glass window, showing the syringe containing the radiopharmaceutical, **d** 1 cm-thick tungsten (W) cylinder used

The syringe is transported to the Nuclear Medicine facilities in a shielded container (Fig. 1a) which contains a cylindrical polymethylmethacrylate (PMMA) tube with the syringe inside (Fig. 1b). Once the nurse is equipped with the dosimeters, the cylinder is extracted from the container and the administration of ^68^Ga-DOTATOC starts, which can be divided into three different steps: the activity control, the injection, and the remaining activity control:

*Step 1:* For the initial activity control, the syringe is extracted from the PMMA cylinder and placed with forceps into the activimeter within the hot cell (Fig. 1c), which is an enclosed, shielded cabinet equipped with an air extraction tube, and front and side doors allowing manual access and the introduction of material to the inside, respectively (Additional file 1: Fig. S1). Once the activity is checked, the nurse shields the syringe with a 1 cm-thick tungsten (W) cylinder (Fig. 1d) and places it on a stainless-steel tray. This step usually takes 60 s.

*Step 2:* The tray with the syringe is transported manually to the patient’s administration room for injection, which is near the hot cell. The radiopharmaceutical is then slowly injected with the syringe shielded, which usually takes 60 s approximately. The injection is performed with an abbocath IV (intravenous) cannula, usually 22 or 24 G, attached to a three-way stopcock and a saline solution to flush the line (Additional file 1: Fig. S2). The syringe with the radioactive material (Fig. 1c) is attached to the three-way stopcock, as well as the saline solution for flushing just after the injection, one to each valve. The third valve from the three-way stopcock is attached to the abbocath IV cannula and injected into the patient. Unlike the other workers, nurse 3 removes the syringe from the tungsten shielding at the time of injection to increase its sensitivity and to administer the drug more comfortably, and once injected, reinserts the syringe back into the shielding.

*Step 3*: After injection, the syringe is returned to the hot cell to check for residual activity in the activimeter, which usually takes 30 s. The syringe is then left in the hot cell for decay.

On average, the whole procedure takes 141 ± 35 s (80–225 s). After 30 min, the patient is positioned for the PET/CT, but no monitoring is done in this step, as it is performed by a different worker, usually a Nuclear Medicine technician.

### Dosimeters and detectors

Doses to extremities, eye lens and whole-body were obtained with two different types of detectors: thermoluminescent detectors (TLDs), which are passive dosimeters, and personal electronic dosimeters (PEDs), i.e., active dosimeters. Each worker was equipped with a personal set of dosimeters, composed of one pair of gloves for hand monitoring, two eye lens detectors, a chest badge for whole-body dosimetry, ring, and wrist dosimeters used in the current clinical practice, and an electronic dosimeter.

**Fig. 2:**
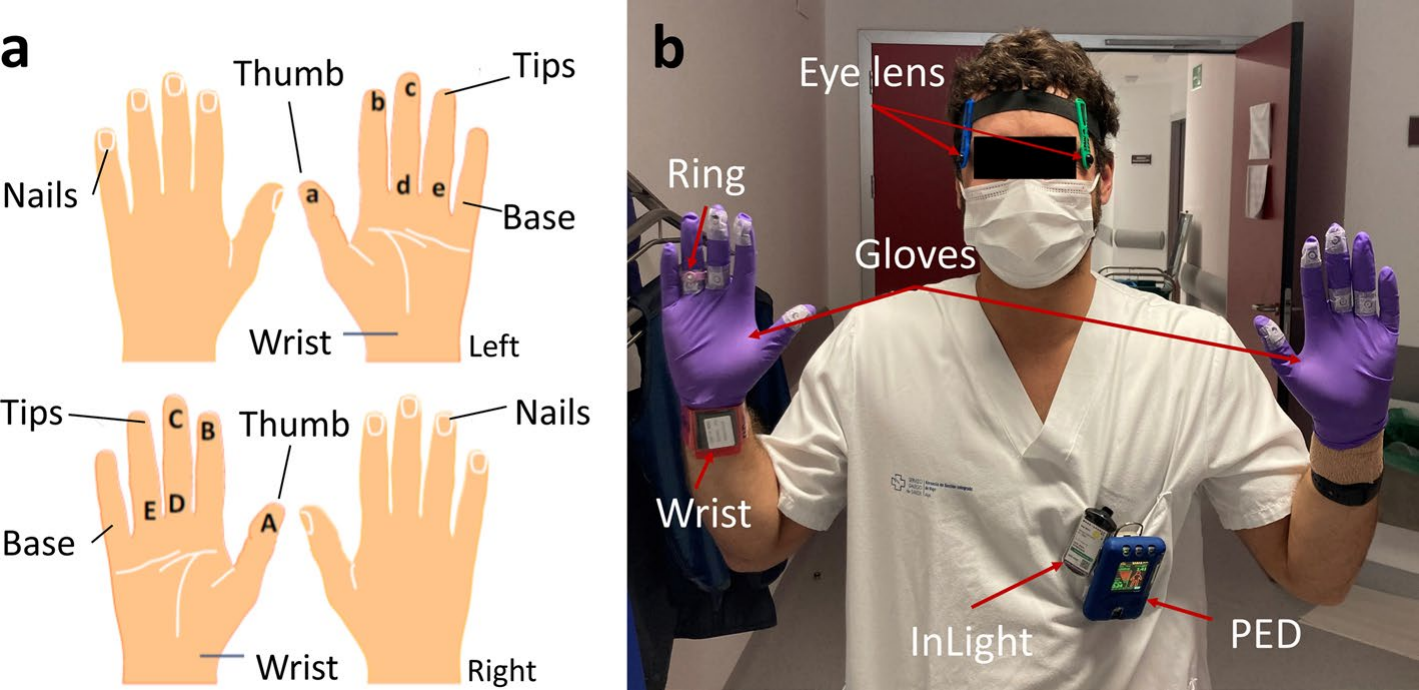
Dosimeters used for staff monitoring: **a** TLD positioning in the hands, **b** all the dosimeters used for extremity, eye lens and whole-body dosimetry

The dose distribution across the hands, in terms of *Hp(0.07)*, was measured with five TLDs per hand attached to a nitrile glove (200 µm-thick nitrile gloves) in several locations, as shown in Fig. 2. These are high-sensitivity TLDs (MCP-Ns (LiF: Mg, Cu, P)) specific for beta radiation, and are provided and analysed by the Belgian Nuclear Research Centre (SCK CEN). These detectors are circular pellets with 4.5 mm diameter and 0.9 mm thickness, consisting of a thin radiation sensitive part (0.5 mm) of 8.5 mg cm^2^ effective thickness, bonded to a thicker, mechanically stable, non-luminescent LiF matrix. They allow reliable measurements of doses in the range of *µSv*, with a detection threshold of 3 µGy [24]. These gloves are covered with regular nitrile gloves to prevent contamination.

In addition to the SCK CEN dosimeters, the personal equivalent dose *Hp(0.07)* was also measured with ring and wrist TLDs (Fig. 2b), which are commonly used for clinical risk appraisal, provided and analysed by the Spanish National Dosimetry Centre (CND). The model of ring dosimeter considered is the TLD DXT-RAD 707H-2 (Thermo Fisher Scientific, Oakwood Village, USA), which is based on a detector of ^7^LiF:Mg,Cu,P, of 7 mg/cm^2^ thickness and 2 mm diameter, glued to a Kapton foil and mounted on an aluminium disc of 4 mm inner diameter and 7 mm outer diameter [25]. This model of dosimeter fulfils the specifications of ISO 12794:2000 [26] and IEC 62,387:2012 [27], allowing for the measurement of *Hp(0.07)* by photons and beta particles in the range of 0.2 mSv–10 Sv [28]. The ring dosimeter is placed at the base of the ring finger of the dominant hand, since this is a common position for workers, with the detector facing the palm side, over the TLD gloves and under the regular nitrile gloves. The CND wrist dosimeter model is the DTX-100, consisting of an anodized aluminium foil with four LiF:Mg,Ti detectors optimized for photons in the range of 0.2 mSv–10 Sv [28]. This model allows for the measurement of *Hp(0.07)* evaluated as the average of the measurement of the four detectors, taking as reference quality that emitted by the ^137^Cs in the case of dosimeters used in nuclear medicine. The wrist dosimeter is located on the dominant hand, facing the detector to the palm side. For both ring and wrist CND detectors, doses below 0.1 mSv are not reported, and deemed as occupational doses.

For monitoring eye doses, specific eye lens dosimeters (EYE-D, Radcard Poland) were used, provided and analysed by SCK CEN. Within the dosimeter, a TLD of type MCP-N (LiF: Mg, Cu, P) was used, allowing for *Hp(3)* measurements in the range of 10 µSv–10 Sv. The SCK CEN dosimetry laboratory is accredited by the Belgian accreditation body BELAC for these TLD measurements in terms of the operational quantities *Hp(3)* and *Hp(0.07)* in the dose range from 50 µSv to 10 Sv according to ISO 17025. In this study, the dosimeters are used within the indications set by the IEC 62,387 norm.

Whole-body dosimetry was performed with active and passive dosimeters. InLight dosimeters (Landauer, Inc., Glenwood, IL) were considered for cumulative (passive) dosimetry, provided, and analysed by SCK CEN. Each worker wore an InLight badge, located at chest level, which is used to measure *Hp(0.07)* and *Hp(10)* quantities. The InLight whole body dosimeter is of the type InLight OSL (Optically stimulated luminescence). This dosimetry system has been approved by the Belgian nuclear control authority (FANC). The dosimeters are sensitive to gamma radiation within the range from 16 keV up to 6 MeV and to beta radiation within the range of 0.7–2.3 MeV. The minimum detection limit is 0.05 mSv. The SCK CEN personal dosimetry service is accredited according to ISO 17025, and the dosimeter conforms the IEC 62387 standard. To complement the cumulative measurements, commercially available personal electronic dosimeters (PEDs) (Tracerco™, London, United Kingdom) located at chest level, allow active dosimetry in terms of equivalent dose *Hp(10)* accumulated in X and gamma radiation fields in the energy range from 33 keV to 1.25 MeV, and at dose rates between 0.1 µSv/h and 100 mSv/h. The dose rate (µSv/h) and effective dose (µSv), both integrated per minute, are recorded for each session.

The above-mentioned detectors (Fig. 2b) are safely stored when no procedures are monitored, with no near radiation sources. The background radiation that the TLDs and InLight whole-body dosimeters receive is determined with an extra InLight and a set of background TLDs for each worker. The lowest detection limit (LDL), that is, the lowest measurable dose, was determined as three times the standard deviation of the background detector.

### Statistical analysis

The cumulative dose from exposure to ^68^Ga-DOTATOC over several sessions for each hand location, both dominant and non-dominant, was normalized to the total activity handled by each worker (GBq), expressed as *Hp*/A. For each location, the mean, standard deviation, and range of this quantity was calculated over all the workers. Based on these values, the Mann–Whitney U test was performed, assuming 95% confidence level, to determine if there were statistically significant differences between doses received at each point between both hands. These calculations were performed with the software R [29]. The same calculations were performed excluding the exposure data from nurse 3 to qualitatively analyse the trend of these metrics when syringe shielding is used.

Following the results obtained in the ORAMED project [6], it is necessary to establish a correction factor (CF) by which to multiply the dose obtained in the different detectors located on the hands in order to correctly determine the maximum dose received. These factors have been defined as the ratio between the dose obtained at the position of maximum exposure and the dose obtained for each of the different detectors distributed on the hands. CFs were obtained individually for each worker and then averaged for all workers.

From real-time measurements, the mean and standard deviation of the maximum dose rate reached (µSv/h) and the cumulative dose (µSv) were computed for each worker over the recorded sessions, as well as the total cumulative dose normalized to the total activity handled (*Hp*/A). These values were averaged over all workers.

Based on these data, annual dose estimations were obtained to determine whether the established dose limits for eye, skin, and effective dose could be exceeded by the product of the maximum dose received by each worker and the estimated activity handled in 1 year.

## Results

### Extremity doses

The range of the dose normalized to the total activity handled (*Hp(0.07)*/A) received in both the dominant (D) and non-dominant (ND) hand for each worker, the site receiving the highest dose (i.e., the most exposed), and the LDL of the TLDs, is outlined in Table 2. The most exposed sites in the non-dominant hand are the thumb (a) (for nurses 1, 5 and 6) and index fingertip (b) (for nurses 2, 3 and 4), whereas in the dominant hand are the index (B) (nurses 1–4) and middle (C) (nurses 5, 6) fingertips. Nurse 4 received the highest dose on the index fingertip (7055 µSv/GBq), followed by nurse 1 (6427 µSv/GBq) on the thumb. In the dominant hand, nurse 3 received the highest dose on the index fingertip (3419 µSv/GBq), which is significantly higher than for the other nurses. *Hp(0.07)*/A values measured with the ring and wrist dosimeters used in routine practice and worn on the dominant hand are also shown for each nurse when the recorded doses were higher than 0.1 mSv.

**Table 2:** Minimum and maximum *Hp(0.07)*/A received in both hands for each nurse. *Hp(0.07)*/A obtained with the CND ring and wrist dosimeters, both worn on the dominant hand, are also shown

Nurse	CND (D) (µSv/GBq)		Gloves						
			ND (µSv/GBq)			D (µSv/GBq)			LDL (µSv)
	Ring	Wrist	Min	Max	Most exposed	Min	Max	Most exposed	
Nurse 1	–	117	848	6427	a	277	966	B	212
Nurse 2	228	76	562	2556	b	199	261	B	212
Nurse 3	474	273	780	3468	b	643	3419	B	212
Nurse 4	–	90	761	7055	b	< LDL	427	B	277
Nurse 5	250	–	710	2107	a	291	677	C	277
Nurse 6	–	400	789	2539	a	< LDL	709	C	92

Unknown values (–) are shown when the dosimeters recorded < 0.1 mSv, so it is not possible to normalize their value to the total activity handled

Table 3 shows *Hp(0.07)*/A values for each position over all workers, and the same calculations excluding the exposure of nurse 3. As expected from the previous results, in the non-dominant hand, the thumb, index, and middle fingertips are the locations receiving the highest doses on average, while it is the index fingertip in the case of the dominant hand in both cases. The dose range monitored over the specific locations of a specific hand is quite large. However, the exclusion of the nurse 3 data causes this dispersion to decrease specifically for the dominant hand, while it remains similar for the non-dominant hand.

**Table 3:** Mean extremity doses normalized to the total activity handled averaged over all the workers (1–6) and excluding the exposure from nurse 3 (1, 2, 4, 5, 6) on both hands

Hand	Location	*Hp(0.07)/*A (Workers 1–6) (µSv/GBq)		*Hp(0.07)/*A (Workers 1, 2, 4–6) (µSv/GBq)	
		Mean ± SD	Range	Mean ± SD	Range
ND	a	2898 ± 1778	1430–6427	3192 ± 1818	2107–6427
	b	2723 ± 1011	1377–3738	2574 ± 1054	1377–3738
	c	2725 ± 2408	849–7055	2802 ± 2683	849–7055
	d	945 ± 223	703–1228	978 ± 232	703–1228
	e	787 ± 155	562–1015	767 ± 164	562–1015
D	A	494 ± 649	< LDL–1583	222 ± 260	< LDL–499
	B	925 ± 1262	< LDL–3419	426 ± 355	< LDL–966
	C	508 ± 372	< LDL–1035	403 ± 300	< LDL–709
	D	257 ± 250	< LDL–643	180 ± 183	< LDL–424
	E	266 ± 255	< LDL–673	184 ± 178	< LDL–394
Ring (D)	E	318 ± 136	228–474	239 ± 15	228–250
Wrist (D)	–	184 ± 135	76–400	171 ± 153	76–400

**Fig. 3:**
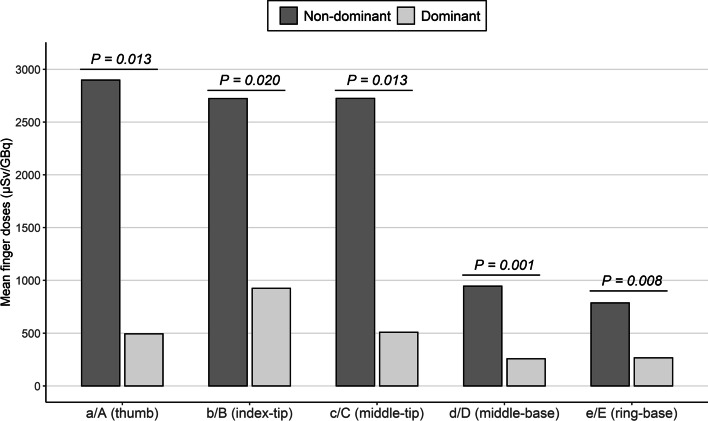
Mean normalized doses on the dominant and non-dominant hands over all workers. The *P* value (*P*) from the Mann–Whitney *U* test is outlined on top of each location

The values for all workers on Table 3 are also depicted in Fig. 3, as well as the *P* value (*P*) from the Mann–Whitney *U*-test performed between the readings of both hands for each location. As can be seen from the data, the mean doses at all locations of the non-dominant hand are higher than those of the dominant hand. According to the Mann–Whitney *U* test, these differences are statistically significant for all locations (*P* < 0.05).

**Fig. 4:**
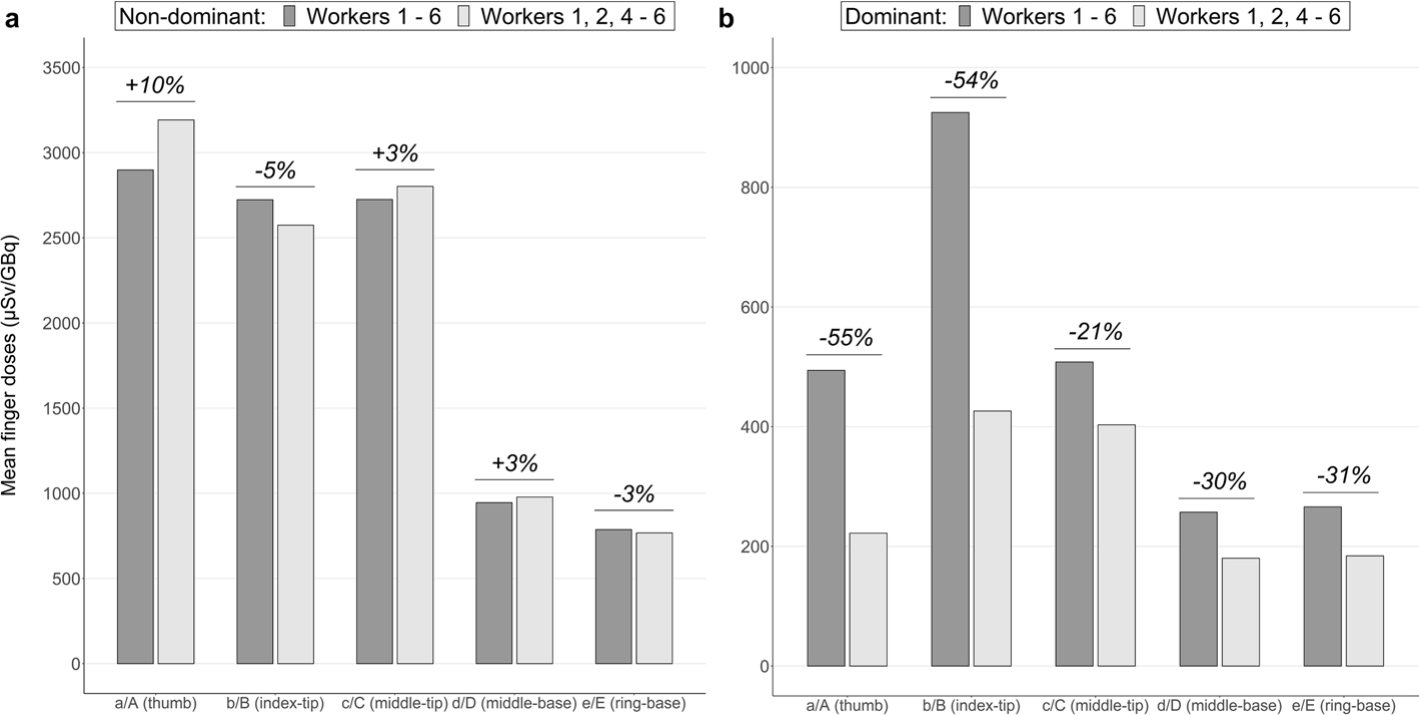
Comparative of the mean finger doses for the **a** non-dominant and **b** dominant hands considering the lectures for all the workers (dark grey) and excluding exposure from nurse 3 (light grey). On top of the bars, the increase (positive percentage) or decrease (negative percentage) on the mean dose when excluding data from nurse 3 is shown

Figure 4 shows the comparison of the averaged equivalent dose *Hp(0.07)*/A for all workers with that obtained by excluding the data from nurse 3 for both the non-dominant (Fig. 4a) and dominant hand (Fig. 4b). The doses received in the non-dominant hand exceed those in the dominant hand in both cases, in agreement with the previous results. Qualitatively, it can be observed that the dose readings at the non-dominant hand positions do not decrease significantly when excluding data from nurse 3, even increasing the thumb exposure by 10%. However, in the dominant hand, they decrease by 54% and 55% at the tip of the index finger and thumb, respectively, as well as by 30% at the bases of the middle and ring fingers, and 20% at the tip of the middle finger.

A CF was calculated for each worker as the ratio between the maximum dose received in the hands and the dose at each of the positions in which it is more common to place the routine dosimeter, that is, the base of the middle finger (d/D) and the base of the ring finger (e/E), in addition to the tip of the index finger (b/B), as performed by Carnicer et al. [30], and the ring and wrist dosimeters. These ratios were first calculated for each worker, and then averaged over all the workers. The range, median and mean values are shown in Table 4. For the three hand positions, the CFs of the dominant hand are higher than those of the non-dominant. CFs of the index tip are nearly 1 in the non-dominant hand, whereas for the base of the index and middle fingers in the non-dominant hand, the median CFs were found to be about 4, and mean values about 5. The ring dosimeter used in this study was placed on the dominant hand, since this is also a common position for workers, obtaining mean and median values about 9. The wrist dosimeter, that was also worn on the dominant hand, present the highest CFs, a median and mean about 34 and 38, respectively.

**Table 4:** Range, median and mean values of the CFs calculated for both hands

Hand	CF (maximum dose/dose at other positions)				
	Base middle (D/d)	Base ring (E/e)	Index tip (B/b)	Ring (CND)	Wrist (CND)
*D*					
Range	5.0–23.2	5.1–16.3	1.0–16.5	7.3–11.2	6.3–78.3
Median	9.1	9.0	6.6	8.4	33.6
Mean	11.6	9.9	7.7	9.0	37.5
*ND*					
Range	1.7–7.8	3.0–9.3	1.0–2.0		
Median	4.0	4.2	1.5		
Mean	4.5	5.0	1.5		

### Eye lens doses

Eye lens monitoring showed *Hp(3)* results below the LDL for all workers (which was 51 µSv for the dosimeters used by nurses 1, 2 and 3; and 43 µSv for nurses 4, 5 and 6) except for the right eye of nurse 2, which received 56 µSv over all the measured sessions, that is, 43 µSv/GBq.

### Whole-body passive and active dosimetry

The whole-body passive dosimeters (InLight) showed *Hp(0.07)* and *Hp(10)* lectures below the LDL for all workers (which was 50 µSv, determined from routine dosimetry protocols) except for nurse 3, who showed 61µSv for *Hp(0.07)* (72 µSv/GBq) and 54 µSv for *Hp(10)* (64 µSv/GBq).

**Fig. 5:**
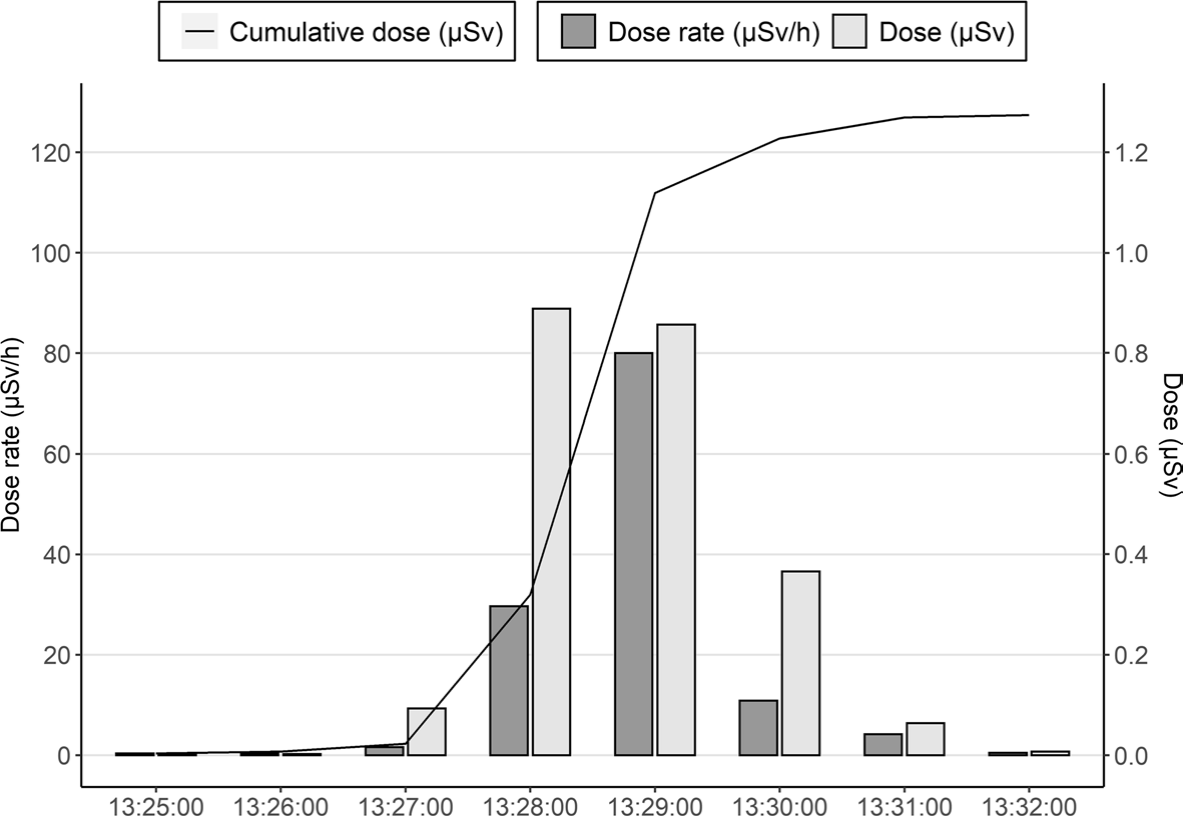
Example of dose rate (µSv/h) and dose (µSv) recorded per minute in one session of ^68^Ga-DOTA-TOC. The process involves the activity check (13:27–13.28), the injection (13:29–13:29) and the post injection activity checking (13:30–13:31). The injection step entails the highest dose rates and doses

Figure 5 shows an example of the dose monitoring in real-time with PED of a nurse administering ^68^Ga-DOTATOC to a patient, in particular nurse 5. As shown in the figure, the maximum dose rate in one minute (µSv/h), the integrated dose per minute (µSv) and the cumulative dose (µSv) are recorded in each session. As seen in the graph, the injection of the radiopharmaceutical (13:28–13:29) entails the highest doses and dose rates.

Table 5 shows the maximum dose rates averaged over all the recorded sessions for each nurse and the total cumulative dose (*Hp(10)*/A), as well as the mean of these values computed over all workers (1–6) and excluding exposure for nurse 3 (no 3), recorded with PEDs. The averaged maximum dose rate is 173 µSv/h per patient, ranging from 79 µSv/h (for nurse 4) to 396 µSv/h (for nurse 3). Excluding data from nurse 3, this value decreases by 21% to 135 µSv/h, the maximum value becoming 265 µSv/h (for nurse 6). In addition, the effective normalized dose was 6.50 ± 2.3 µSv/GBq. Nurse 3 reported the highest effective normalized dose (9.9 µSv/GBq), so by excluding this measurement, the dose value decreases by 11% to 5.81 ± 1.77 µSv/GBq.

**Table 5:** Mean, SD and range of the maximum dose rate values recorded with PEDs in each session and the cumulative dose normalized to the total activity handled

Worker	Dose rate (µSv/h)	*Hp(10)/*A (µSv/GBq)
Nurse 1	110 ± 52 (51–195)	5.2
Nurse 2	220 ± 83 (125–349)	8.8
Nurse 3	396 ± 202 (194–677)	9.9
Nurse 4	79 ± 18 (57–110)	4.6
Nurse 5	109 ± 22 (89–150)	6.1
Nurse 6	265 ± 0 (265–265)	4.4
Mean (1–6)	173 ± 134	6.5 ± 2.3
Mean (no 3)	135 ± 76	5.8 ± 1.8

### Annual dose estimations

Annual dose estimations were performed assuming that 80 patients are imaged each year with 200 MBq of ^68^Ga-DOTATOC in our hospital, as this is a common patient rate. Assuming that the patient workload is equally distributed, each worker would be performing about 14 procedures. Therefore, the total activity handled per year and per worker can be estimated at 3 GBq.

Table 6 shows the annual skin dose and annual effective dose estimations performed for each worker, the former based on the measured normalized maximum dose (Table 2), as performed in the ORAMED study [6] and the latter based on the effective doses recorded for each worker with PEDs (Table 5). In addition, an estimation for *Hp(10*)/A was made from the value recorded with the InLight dosimeter for nurse 3.

**Table 6:** Annual *Hp(0.07)* estimations for extremities based on the maximum dose recorded with TLD gloves, and annual *Hp(10)* estimations for whole-body based on the total cumulative dose recorded with PEDs and the InLight dosimeter in case of nurse 3

Worker	Max. *Hp(0.07)*/A (µSv/GBq)	Annual *Hp(0.07)* estimate (mSv)	*Hp(10)*/A (µSv/GBq)	Annual *Hp(10)* estimate (mSv)
Nurse1	6427	19	5.2	0.02
Nurse2	2556	8	8.8	0.03
Nurse3*	3468	10	9.9/64	0.03/0.20
Nurse4	7055	21	4.6	0.01
Nurse5	2107	6	6.1	0.02
Nurse6	2539	8	4.4	0.01

*Showing both *Hp(10)* obtained with PED and InLight

As seen in Table 6, the annual equivalent dose *Hp(0.07)* estimated to the hands ranges from 6 to 21 mSv, corresponding to nurse 5 and 4, respectively. In addition, the estimated annual effective doses *Hp(10)* recorded with the active dosimeters range from 0.01 to 0.03 mSv, whereas according to the InLight measurements, nurse 3 is estimated to reach an effective dose of 0.2 mSv/year, which is an order of magnitude higher than the estimation with PEDs.

The doses recorded with the eye lens dosimeters showed 43 µSv/GBq for the right eye of nurse 3, whose annual dose would be estimated as 0.15 mSv/y. Since the rest of the TLDs showed readings below this value, in this study this is the maximum estimation for the eyes.

## Discussion

The aim of this study was to investigate the radiation exposure of the skin of the hands, eye lenses and whole body of nuclear medicine workers with multiple measuring devices during the manipulation of ^68^Ga-DOTATOC for diagnosis. This study would contribute to the standardization of the protocols followed during these novel procedures, minimising the radiation exposure of the staff.

In this study we showed that during the administration of ^68^Ga-DOTATOC, the non-dominant hand is more exposed than the dominant hand, being the thumb, the index and middle fingertips the sites receiving higher doses in the former. These results are in good agreement with other studies for other radiopharmaceuticals, such as the ORAMED study [6, 30, 31] which states that the non-dominant hand usually receives higher doses than the dominant hand, since it is the hand that holds the syringe or vial, and the index tip followed by the thumb of the non-dominant, are the most exposed sites for almost all procedures. The fingertips receiving the highest doses is also confirmed in many more studies [14, 30–32].

The exposure data recorded with the TLDs on the gloves were compared with those obtained with the official ring and wrist dosimeters used for routine practice monitoring. As seen in Table 2, the maximum *Hp(0.07)*/A values measured with the ring dosimeter, when possible, are significantly lower than the maximum doses recorded with the glove’s TLDs of the non-dominant hand, while it is not that acute compared to the dominant hand, except for the values recorded in nurse 3. In addition, the averaged normalized dose values recorded with the wrist and ring dosimeters, showed in Table 3, also remain below the highest averaged doses. However, the TLD placed on the base of the dominant hand and the official ring dosimeter (E) showed similar results (266 µSv/GBq and 318 µSv/GBq), which is a good confirmation of the trustworthiness of the monitoring protocol and the suitability of the CND detectors. Nevertheless, this underestimation is more severe with the wrist dosimeter.

It has been quantitatively shown that when the exposure data from nurse 3 are included, there is a significant increase in the mean doses of the dominant hand, while remaining similar in the case of the non-dominant hand. This result, coupled with the fact that nurse 3 recorded significantly higher doses than the other nurses in the dominant hand, and that the dose range decreases especially on this hand when their data are omitted, indicate that when no shielding is used over the syringe during the ^68^Ga-DOTATOC injection, the exposure of the dominant hand may increase more severely than in the non-dominant hand.

The CFs obtained on the index fingertip on the non-dominant hand were found to be almost one, which is in good agreement with the previous results, as this means that it is one of the most exposed locations. Therefore, if this location was used to monitor these procedures, there would be practically no need to apply a CF to estimate the maximum doses. However, since placing a dosimeter on the tip of the index finger may hamper work, the base of the middle and ring fingers of the non-dominant hand may be a suitable position, as they present lower mean and median values than other locations. For these positions, the median and mean CFs were found to be about 4 and 5, respectively, which are in the same order of magnitude than those reported from other studies, such as Carnicer et al. within the ORAMED project [30] who reported a CF of 6 for the index base for all the procedures considered, or Wrzesien et al. [32] obtaining a CF about 4 for the middle finger of the non-dominant hand for ^99m^Tc procedures. Based on these results, at least a CF of 5 may be chosen to account for the maximum dose received in the hands to avoid the risk of underestimation.

Placing the CND ring dosimeter on the dominant hand leads to higher CFs values for this dosimeter, but similar values are obtained to the TLD placed on the same position (E), which also supports the suitability of the official ring dosimeters in these cases. The mean and median CFs are significantly higher for the CND wrist dosimeter, which indicates that the use of wrist dosimeters for these procedures may severely underestimate the maximum doses received on the most exposed fingers, leading to the recommendation of using a ring dosimeter instead. This is in compliance with the ORAMED study, which recommended not to use wrist dosimeters for estimating nuclear medicine doses, as they showed a poor correlation and a high risk of underestimation [6, 30].

Personal equivalent doses *Hp(3)* for the eyes did not result in values above the LDL for almost all workers, except for nurse 2 (43 µSv/GBq). The authors found one study by Wrzesień et al. [33] regarding occupational eye lens doses from procedures involving ^68^Ga-DOTATATE, which despite being a different compound is appropriate for comparison. They found that the maximum *Hp(3)*/A recorded value was over 128 µSv/GBq in the group of nurses performing the injection, which is higher than the value obtained in this study. However, they obtained mean equivalent dose values about 64 and 47 µSv/GBq to the right and left eye, respectively, which is a similar result to the value obtained in this study for nurse 2. On the one hand, this indicates that the dosimeters used in this study are suitable for eye lens dose monitoring but that it is necessary to accumulate the dose over more sessions in order to obtain measurable values. On the other, that more measurements are needed to make an adequate assessment, as there may be large variabilities between workers and sites.

The whole-body passive dosimeters showed values below the LDL except for nurse 3. This increase may be due to the lack of shielding at the time of injection, which reinforces the recommendation to shield the syringe. However, this value showed disparity with the effective doses recorded with PED, being 64 µSv/GBq and 9.9 µSv/GBq, respectively. This significant difference is possibly due to the nonlinear response of the Geiger-Müller detector at low energies, which is the basis of the PED used in this study. Since the InLight dosimeters used were specifically chosen to measure the ^68^Ga beta radiation, it is more likely that the effective doses recorded with PEDs are underestimated. Nevertheless, this discrepancy may be thoroughly investigated to corroborate the suitability of whole-body dosimeters during these procedures.

In addition, from the real-time data measured with PEDs, it is shown that the highest dose rates are recorded during the injection of the radiopharmaceutical, which also reinforces the recommendation of using syringe shielding during this step. The values of dose rates obtained in this study (Table 5), are in good agreement with the results shown by Portela et al. [17] for ^68^Ga-DOTATOC, whose study reported maximum and minimum values from one worker about 400 µSv/h and 80 µSv/h, respectively. Therefore, although real-time PEDs may underestimate effective doses, they might be valuable to record dose rates.

Finally, annual dose estimations were made. It has been obtained that neither the eye lens, hands nor the whole-body dose estimates will exceed the annual dose stablished by European law 2013/59/Euratom [34] from the management of ^68^Ga-DOTATOC for PET imaging, which is in good agreement with other studies dealing with positron-emitting radiopharmaceuticals [14, 35] in which it is shown that it is highly unlikely to exceed the annual dose limits. Nevertheless, it should be considered that the values obtained for *Hp(10)*/year from PED may be underestimated, so the 0.2 mSv/year estimate from nurse 3 may be a more realistic approximation, despite not using syringe shielding during injection. However, one worker will usually perform several procedures involving multiple isotopes, so the real situation is more complex. Some of the most commonly used radioisotopes for diagnosis are ^99m^Tc and ^18^F. The ORAMED study [6] determined the range, mean and median of the maximum normalized doses received to the hands during the preparation and administration of both radiopharmaceuticals, as seen in Table 7, so the relative impact of ^68^Ga with respect these radioisotopes can be done by calculating the same parameters of the maximum normalized dose from *Hp(0.07)/A* data shown in Table 2 for each nurse. As seen in Table 7, the administration of ^68^Ga entails the highest dose values compared to ^18^F and ^99m^Tc. The latter is a gamma emitter (photons of 140 keV (87%)), so it is reasonable that the dose to the hands is higher for positron-emitter radionuclides, such as ^18^F and ^68^Ga. In addition, the positrons emitted during the decay of ^18^F (maximum *β*+ energy 634 keV (96%)) are less energetic than those from ^68^Ga, so the range of the positrons is shorter [36] and therefore, the dose recorded with the dosimeters during ^68^Ga administration is higher. Moreover, the dosimeters used in the ORAMED project were not the high-sensitive positron detectors that were used in this study [6] which can also explain the higher doses measured with ^68^Ga. Nevertheless, although the normalized doses are higher for ^68^Ga, currently the number of patients diagnosed with ^18^F and ^99m^Tc exceed the number of patients diagnosed with ^68^Ga. Therefore, although the dose received to the hands, which is the most exposed part of the body due to the positron emission, might not be relatively as high as the dose received with other radiopharmaceuticals, care must be taken to ensure that increase in the number of patients diagnosed with ^68^Ga does not lead to an exceedance of the annual dose limits by working with several isotopes.

**Table 7:** Maximum normalized extremity dose values in mSv/GBq for ^99m^Tc and ^18^F, obtained from the ORAMED project [6], and the same data for ^68^Ga obtained from this study, inferred from Table 2

Procedure	Maximum normalized dose (mSv/GBq)		
	Range	Mean	Median
^99m^Tc administration	0.01–0.95	0.23	0.12
^18^F administration	0.14–4.11	0.93	0.64
^68^Ga administration	2.11–7.06	4.03	3.01

## Limitations and future work

The study was limited to the results obtained from six nurses monitored over a total of 28 sessions. However, given the large variabilities between data from different nurses, as also reported by other authors [13, 30, 37], providing a larger sample size would increase the statistical power, especially reducing the uncertainties in the CF estimation. In future measurements, the CND ring dosimeter will be placed on the non-dominant hand to correctly corroborate the validity of the CF values obtained in this study specifically on these dosimeters. Moreover, further measurements are needed to compare the effective dose *Hp(10)* recorded with passive and active dosimeters to verify the suitability of the latter for these procedures. Another major limitation is that there are insufficient data to quantitatively compare the doses received by nurses using and not using syringe shielding, as only one of the nurses performed this practice. However, after the completion of this study the nurse now uses the shielding to inject the drug, and for the sake of their safety injecting without shielding will not be performed again, so it is not possible to continue to observe this trend. Moreover, this study was focused on the administration step, but it would be of great interest to extend the research to all stages involving the manipulation of ^68^Ga-DOTATOC, from its synthesis in ^68^Ga/^68^Ge generators, as performed by Dwivedi et al. [16] for [^68^Ga]Ga-DOTA-NOC, to the positioning of the patient in the PET scanner, which as mentioned before, although performed by a Nuclear Medicine Technician, it constitutes another source of radiation that should be interesting to take into account. Finally, the risk of potential contamination has not been addressed in this study. Although skin contamination is prevented by using disposable gloves, its assessment is crucial in Nuclear Medicine, especially when dealing with positron or beta emitters, such as ^68^Ga. Therefore, as future work, it would be interesting to account for the skin dose or the effective dose due to inhalation and/or ingestion of the radiopharmaceutical.

The authors are currently continuing to monitor the workers involved in gallium diagnostics to further extend the scope of this study. Simultaneously, nuclear medicine personnel involved in [^177^Lu]Lu-DOTA-TATE treatments are being monitored with similar detectors with the aim of performing dosimetry studies of theranostic procedures with the tandem [^68^Ga]Ga-DOTA-TOC / [^177^Lu]Lu-DOTA-TATE.

## Conclusions

This study aims to address the scarcity of dosimetric studies in nuclear medicine personnel, with the object of standardising procedures during the handling of new radiopharmaceuticals, particularly of ^68^Ga-DOTATOC, thus reducing variations in dosimetric methods for the determination of the maximum doses received by workers. For this purpose, extremity, eye lens and whole-body doses in terms of personal equivalent doses, *Hp(0.07)*, *Hp(3)*, and personal effective dose *Hp(10)*, respectively, were assessed with thermoluminescent (passive) and electronic dosimeters (active) to six nurses administering ^68^Ga-DOTATOC for diagnostics with PET.

It has been concluded that the non-dominant hands are more exposed to radiation than the dominant hands. It was also shown that the use of a syringe shield during the injection step is needed to reduce the dose to the hands, especially for the dominant hand. The most exposed sites within the non-dominant hand are the thumb and the index fingertip, whereas the index fingertip is the most frequently exposed site from the dominant hand.

Wrist dosimeters, used in the clinical practice, largely underestimate maximum doses, as well as the ring dosimeters if they are placed on the base of the fingers of the dominant hand. However, placing a ring dosimeter at the bases of the index or middle finger of the non-dominant hand will provide a good estimation of the maximum dose if a conversion factor of 5 is applied.

Finally, it has been shown that although it is very unlikely that annual dose limits are exceeded for all monitoring position, care must be taken to ensure that an increase in the number of patients diagnosed with ^68^Ga does not lead to a significant increase in the annual dose when compared also to other radiopharmaceuticals.

## Supplementary Information

**Additional file 1:** The available supplementary materials show additional details about the nuclear medicine facilities and materials used for the administration of ^68^Ga-DOTATOC.

## Data Availability

The datasets used and analysed during the current study are available from the corresponding author on reasonable request.

## Abbreviations

^137^Cs: Caesium-137
^177^Lu: Lutetium-177
^18^F: Fluorine-18
^68^Ga: Gallium-68
^68^Zn: Zinc-68
^90^Y: Yttrium-90
^99m^Tc: Technetium-99 metastable
CND: Spanish National Dosimetry Centre
CT: Computed tomography
DOTANOC: DOTA-NaI3-Octreotide
DOTATATE: DOTA-Tyr3-Octreotate
DOTATOC: DOTA-Phe1-Tyr3-Octreotide
EANM: European Association of Nuclear Medicine
FDG: Fluorodeoxyglucose
GEP: Gastroenteropancreatic
LDL: Lowest detection limit
NET: Neuroendocrine tumours
NM: Nuclear Medicine
OSL: Optically stimulated luminescence
PCa: Prostate cancer
PED: Personal electronic dosimeters
PET: Positron emission tomography
PMMA: Polymethyl methacrylate
PRRT: Peptide receptor radionuclide therapy
RIT: Radioimmunotherapy
SCK CEN: Belgian Nuclear Research Centre
SD: Standard deviation
SSTR: Somatostatin receptor
TLD: Thermoluminescent dosimeters
W: Tungsten
