# Supplementary material for: Occupational radiation exposure assessment during the management of [68Ga]Ga-DOTA-TOC
Source: EJNMMI Phys. 2022 Oct 29;9:75. doi: 10.1186/s40658-022-00505-8 (PMC9617990; doi:10.1186/s40658-022-00505-8)
Supplement: Supplementary file 1 — Additional file 1: The available supplementary materials show additional details about the nuclear medicine facilities and materials used for the administration of 68Ga-DOTATOC. [file 40658_2022_505_MOESM1_ESM.docx]

**Supplementary information**

**Fig. S2** Injection set up. (A) syringe and three-way stopcock. (B) abbocath IV cannula attached to the three-way stopcock and the butterfly needle, which is not used due to its longer length, which may result in more residual activity remaining in the catheter, even after flushing.


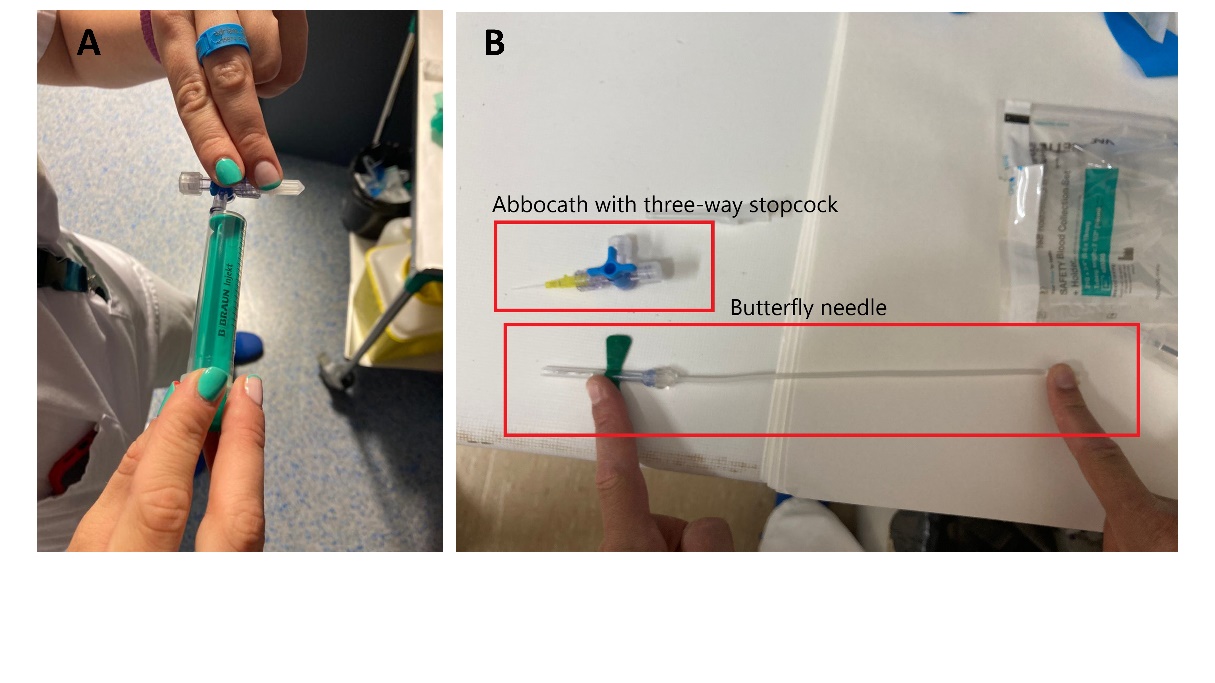

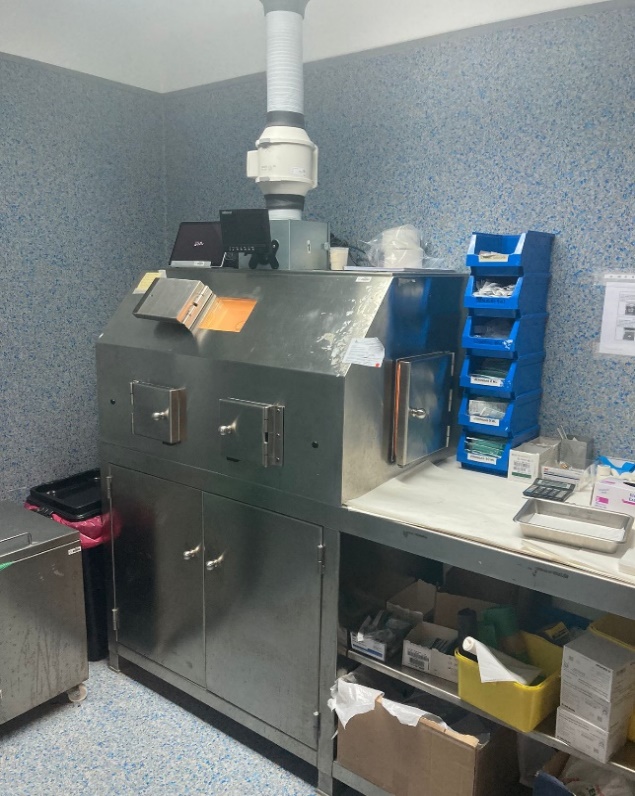


**Fig. S1** Exterior of the structure (hot cell) inside which the activity of the radioactive material is measured. Its interior is shown in Fig.1c of the manuscript through the lead glass window.
